# Virtual noncontrast images of adrenal lesions: a photon-counting CT prospective study

**DOI:** 10.1186/s41747-025-00621-x

**Published:** 2025-08-29

**Authors:** Xin Bai, Lin Lu, Anli Tong, Jianhua Deng, Lili Xu, Xiaoxiao Zhang, Jiahui Zhang, Li Chen, Qianyu Peng, Erjia Guo, Yongfei Wu, Yun Wang, Kai Xu, Chao Zhang, Xi Zhao, Zhengyu Jin, Gumuyang Zhang, Hao Sun

**Affiliations:** 1 Department of Radiology, State Key Laboratory of Complex Severe and Rare Disease, Peking Union Medical College Hospital, Peking Union Medical College, Chinese Academy of Medical Sciences, Beijing, China; 2 Department of Endocrinology, State Key Laboratory of Complex Severe and Rare Disease, Peking Union Medical College Hospital, Peking Union Medical College, Chinese Academy of Medical Sciences, Beijing, China; 3 Department of Urology, Peking Union Medical College Hospital, Peking Union Medical College, Chinese Academy of Medical Sciences, Beijing, China; 4 Department of Radiology, Zhejiang Cancer Hospital, Hangzhou Institute of Medicine (HIM), Chinese Academy of Sciences, No. 1, East Banshan Road, Gongshu District, Hangzhou, Zhejiang China; 5 Siemens Healthineers Digital Technology (Shanghai) Co. Ltd., Shanghai, China

**Keywords:** Adenoma, Adrenal gland diseases, Adrenocortical adenoma, Tomography (x-ray computed)

## Abstract

**Background:**

The value of virtual noncontrast (VNC) images from photon-counting computed tomography (PCCT) for evaluating adrenal lesions and diagnosing adrenal adenomas remains to be clarified.

**Materials and methods:**

Participants with adrenal masses who underwent unenhanced and portal venous phase PCCT were prospectively included. Portal-venous phase images were reconstructed using conventional VNC (VNC_Conv_) and PureCalcium VNC (VNC_PC_). We measured two-dimensional (2D) attenuation of adrenal masses at their largest slice on true noncontrast (TNC), VNC_Conv_, and VNC_PC_ images. Three-dimensional (3D) attenuation and radiomic features of adrenal masses were semiautomatically extracted. These parameters were statistically compared, and diagnostic performance for adenomas was evaluated.

**Results:**

The study included 54 participants (27 females, mean age 45.3 years) with 68 adrenal lesions. Attenuation values on VNC were higher than those on TNC. TNC, VNC_Conv_, and VNC_PC_ attenuation values did not differ between 2D and 3D measurements. The intraclass correlation coefficients of first-order, shape, and texture features between TNC and VNC were 0.671, 0.822, and 0.616, respectively. The sensitivity and specificity of the proposed thresholds (VNC_Conv_ 25 HU, VNC_PC_ 20 HU) were higher than those of the previously established threshold of 10 HU in diagnosing adenomas. There was no significant difference between VNC_Conv_ and VNC_PC_ in diagnosing adenomas (area under the receiver operating characteristic curve: 0.841 *versus* 0.838, *p* = 0.873).

**Conclusion:**

VNC algorithms from PCCT overestimated CT attenuation of adrenal lesions. Higher thresholds showed better diagnostic performance for discriminating adrenal adenomas from non-adenomas than the established 10 HU.

**Relevance statement:**

We investigated the application of VNC images from PCCT in adrenal disease. On VNC images, higher thresholds, superior to the accepted 10 HU, are needed for discriminating adenomas from non-adenomas, reducing the need for secondary examinations.

**Key Points:**

- This study investigated the value of VNC images from PCCT in adrenal lesions.
- VNC reconstruction overestimated the CT attenuation of adrenal lesions.
- Higher thresholds on VNC images were superior to the accepted 10 HU for differentiating adenomas from non-adenomas.

**Graphical Abstract:**

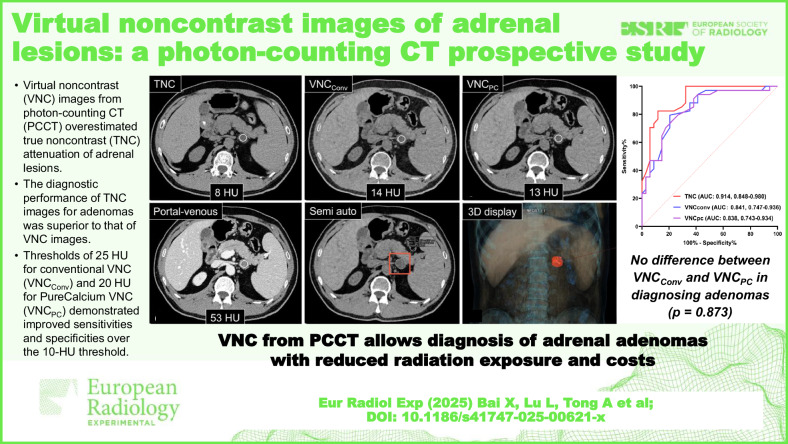

## Background

Adrenal adenomas are the most common adrenal lesions, accounting for 50–80% of cases [1]. Approximately 70% of adrenal adenomas contain abundant intracellular fat and can be diagnosed as lipid-rich adenomas based on unenhanced attenuation values (≤ 10 HU) [1, 2], whereas almost all non-adenomas are deficient in intracellular fat and exhibit higher computed tomography (CT) attenuation [1]. Therefore, unenhanced CT as a lipid-sensitive imaging technique has been found to be highly effective at distinguishing adenomas and non-adenomas [1]. A threshold of 10 HU has been established for the diagnosis of adrenal adenomas, with sensitivities ranging 71–79% and specificities ranging 96–98% [3, 4].

Adrenal lesions are often indeterminate on single-phase contrast-enhanced CT due to their considerable overlap in attenuation, which may pose a diagnostic challenge [1, 5]. Dual-energy CT (DECT) represents an effective solution to this problem, as it enables virtual noncontrast (VNC) images to be generated from single contrast-enhanced images [6]. Several previous studies have explored the feasibility of VNC reconstruction for evaluating adrenal lesions [6–10], and these studies all suggested that VNC attenuation based on portal venous phase DECT tended to overestimate adrenal lesion attenuation.

Photon-counting CT (PCCT) is a new type of CT scanner that uses a direct conversion x-ray detector that provides improved spatial resolution, iodine signal, and dose efficiency [11], enabling virtual monoenergetic images and material classification that support numerous diagnostic tasks in abdominal imaging [11–13]. Recent studies compared the CT attenuation of VNC reconstructions from PCCT with true noncontrast (TNC) images for evaluating adrenal lesions [14, 15]. However, CT attenuation on VNC images from PCCT tended to be underestimated or overestimated, but the reasons for this were not investigated.

This study compared CT attenuation between VNC images derived from different VNC algorithms with TNC images of adrenal lesions from two-dimensional (2D) and three-dimensional (3D) PCCT data. The potential reasons for their differences were explained from the perspective of radiomic features, and the optimal threshold of CT attenuation for differentiating between adrenal adenomas and non-adenomas was determined.

## Materials and methods

### Study design and participants

**Fig. 1:**
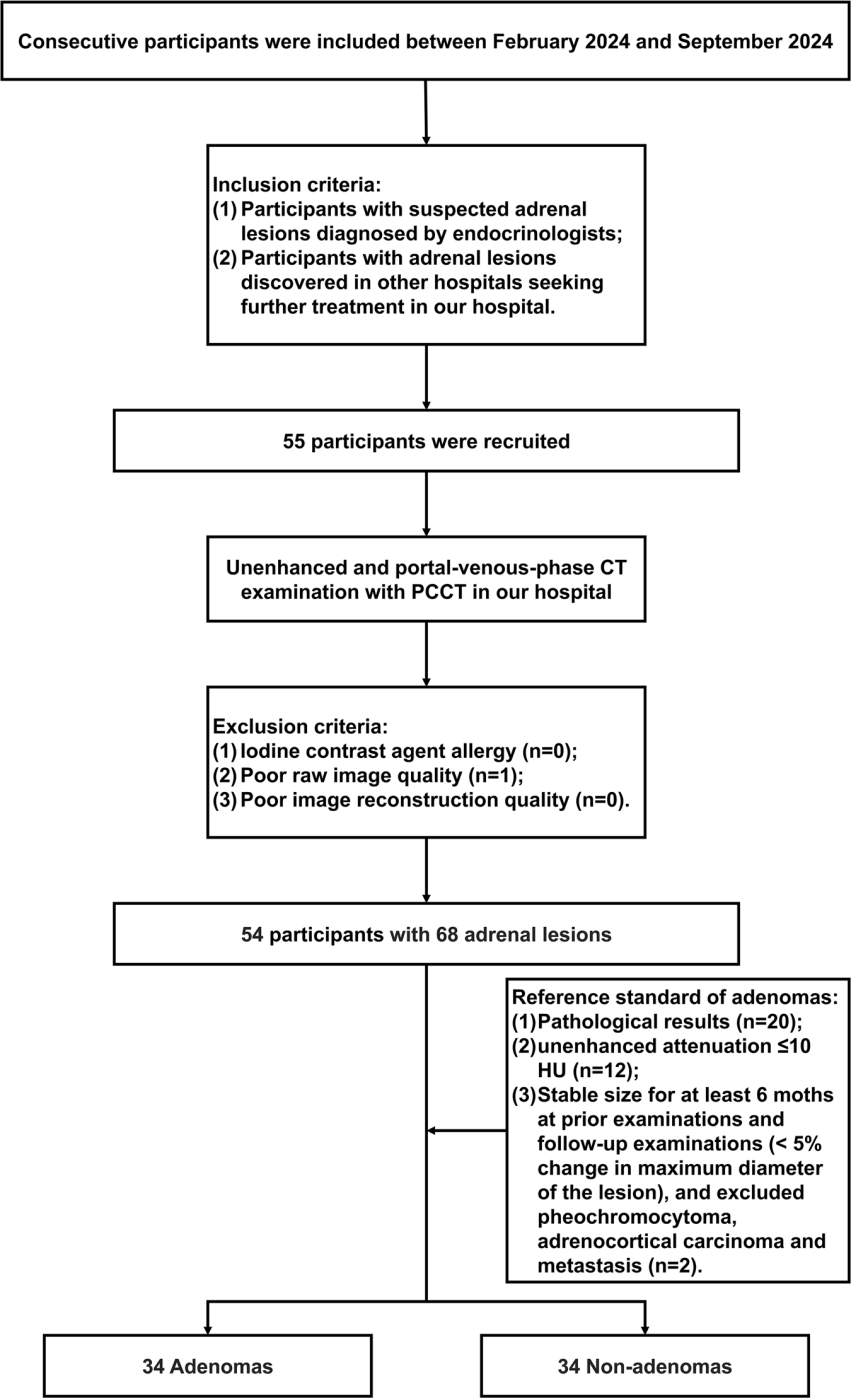
The study flow diagram of participant inclusion and exclusion

This prospective study was approved by the Institutional Review Board of our hospital (no. I-23PJ1487), and informed consent was received from all participants. Participants were consecutively enrolled between February 2024 and September 2024. Inclusion criteria were as follows: (1) participants with suspected adrenal lesions diagnosed by endocrinologists; and (2) participants with adrenal lesions discovered at other hospitals and seeking further treatment in our hospital. Participants who met the inclusion criteria underwent unenhanced and portal venous phase CT examination with PCCT in our hospital. Exclusion criteria were as follows: (1) iodine contrast agent allergy; (2) poor raw image quality; or (3) poor image reconstruction quality (Fig. 1).

### CT protocols

All participants underwent CT examinations using a dual-source PCCT (NAEOTOM Alpha, Siemens Healthineers). The participants were placed in the supine position with a scan range from above the hemidiaphragm to the pubic symphysis. The scanning parameters were as follows: 120 kVp tube voltage, variable tube current with automatic tube current modulation activated (CARE Dose4D), 144 × 0.4 mm^3^ collimation, 0.5-s rotation, 0.8 pitch. After unenhanced image acquisition, 80 mL of nonionic contrast agent (Ultravist 370, Bayer AG) was injected at 4 mL/s, with an iodine flux of 1.5 g/s. Arterial phase images were acquired 7 s after reaching the bolus-triggering threshold of 120 HU at the thoracoabdominal aortic junction. Portal venous phase images were acquired 45 s after the arterial phase.

### Image reconstruction

Image reconstruction was performed from image raw data using dedicated research software (syngo.via CT VA50A, Siemens Healthineers). VNC images were reconstructed from portal venous phase CT images using two different algorithms, conventional VNC (VNC_Conv_) and PureCalcium VNC (VNC_PC_). The reconstruction parameters were as follows: axial view; 1-mm section thickness; 0.7-mm section intervals; Qr40 kernel, 512 × 512 matrix; quantum iterative reconstruction strength, four; spectral reconstruction, VNC_Conv_, or VNC_PC_.

### Image analysis

**Fig. 2:**
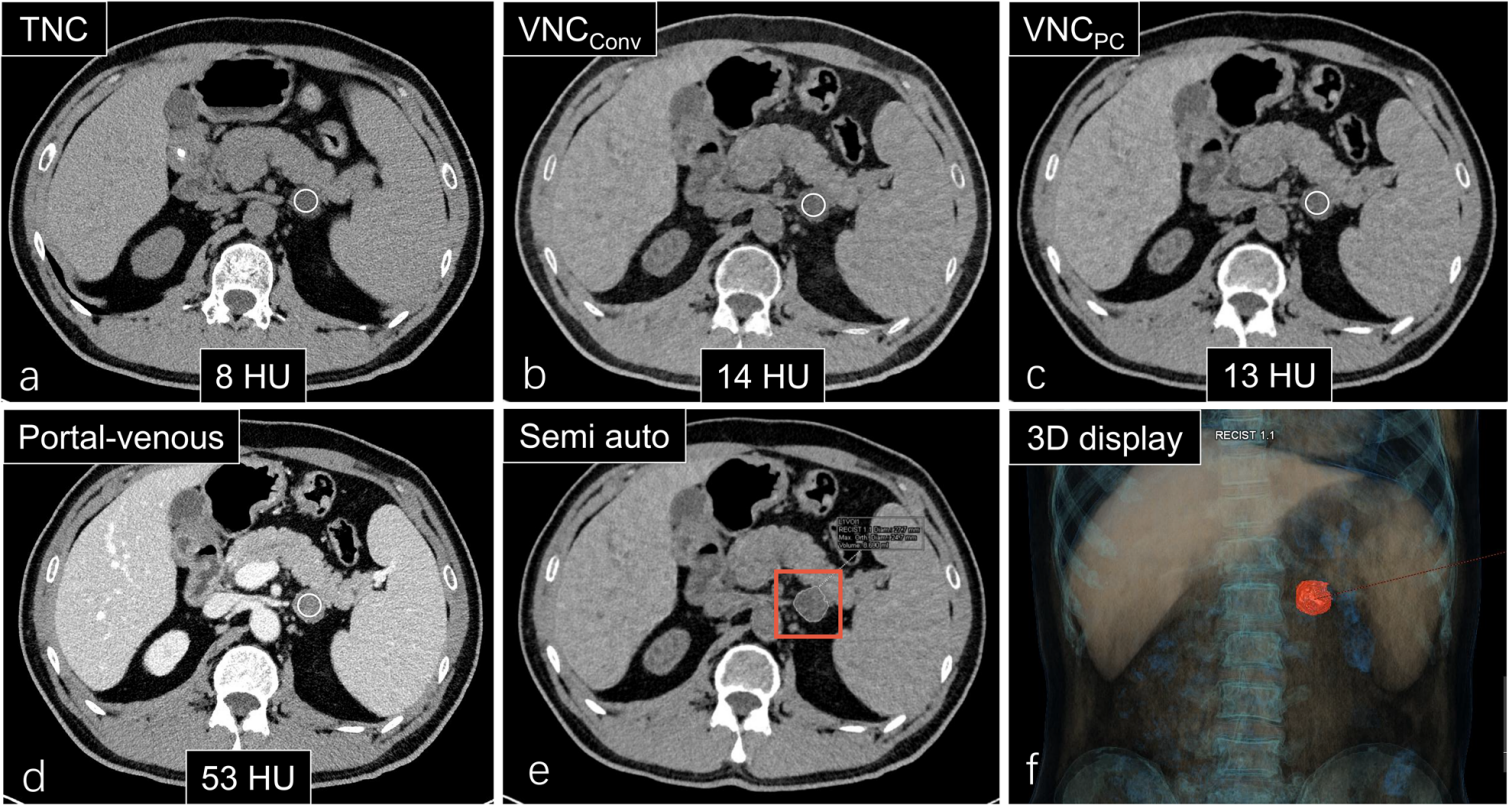
Overview of the methodology. A 45-year-old female with a left adrenal mass with a pathological diagnosis of adenoma. **a** Attenuation on TNC; **b** attenuation on VNC_Conv_; **c** attenuation on VNC_PC_; **d** attenuation on the portal venous phase; **e** semiautomatic segmentation of the adrenal mass; and **f** 3D display of the adrenal mass. TNC, True noncontrast; VNC, Virtual noncontrast; VNC_Conv_, Conventional VNC; VNC_PC_, PureCalcium VNC

Image analysis was achieved using commercial software from Siemens Healthineers (version: syngo.via CT VB70A and VB20A). For 2D measurements, we measured CT attenuation of adrenal masses at the largest slice on TNC, VNC_Conv_, and VNC_PC_ images. Semiautomatic segmentations of adrenal lesions were performed by a radiology resident with 5 years of experience (X.B.) to extract 3D CT attenuation of entire lesions from TNC and VNC images. Additionally, we analyzed the agreement between TNC and VNC radiomic features of lesions, including first-order, shape, and texture features, using syngo.via Frontier Radiomics software (version: 1.4.0) from Siemens Healthineers CT (Fig. 2). The TNC images were imported into the software, and the long diameter of the lesion was delineated on the axial images to semi-automatically segment the lesion (*i.e.*, mask). The radiomics features were automatically extracted by the software, and the radiomics feature values were derived. Meanwhile, the mask was copied into the corresponding VNC image to obtain the radiomics feature values from the VNC image as well.

### Reference standard of adenomas

We recognized adenomas according to the following criteria: (1) pathologically confirmed adenoma; (2) no pathological results but TNC attenuation ≤ 10 HU [7, 14]; and (3) no pathological results and TNC attenuation > 10 HU, but stable tumor size for at least 6 months at prior examinations and follow-up examinations (< 5% change in maximum diameter of the lesion) [14] and endocrine hormone tests excluded pheochromocytoma and adrenocortical carcinoma; absence of history of malignancy excluded metastasis. All participants completed at least 6 months of imaging follow‑up.

### Statistical analysis

Statistical analyses were performed using SPSS (version 27.0, IBM) and GraphPad Prism (version 8.3.0, GraphPad Software). Normality was first evaluated using the Shapiro-Wilk test. Normally distributed continuous data were described using mean ± SD. Categorical variables were expressed as counts and percentages. Normally distributed continuous variables were compared using paired *t* tests and one-way ANOVA. Categorical variables were compared using the χ^2^ test. Bland–Altman analysis was performed to assess agreement between TNC and VNC attenuation. Pearson correlation analysis and linear regression analysis were used to show the correlations between TNC and VNC. The intraclass correlation coefficient (ICC) was calculated to show the agreement between radiomic features on TNC and VNC_Conv_ images. The ICC was classified as an excellent agreement if the ICC was greater than 0.80. Bonferroni correction was applied for multiple tests of ICCs of radiomics features (*p* < 0.05 [α]/1,598 [number of tests], *i.e.*, *p* < 3.13 × 10^−5^). Receiver operating characteristic (ROC) curves were generated, and the area under the ROC curve (AUC), sensitivity, specificity, and thresholds were calculated. AUC comparisons were performed with the DeLong test. Post-hoc power analysis was conducted to confirm the adequacy of the sample size. A two-sided *p*-value < 0.05 was considered statistically significant.

## Results

### Participant characteristics

A total of 55 participants were included according to the inclusion criteria and underwent unenhanced and portal venous phase CT examination with PCCT in our hospital. One participant was excluded because of poor image quality. The final study cohort consisted of 54 participants (27 female, mean age 45.3 years [range 17–69]) with 68 adrenal lesions (Fig. 1). Fourteen participants had bilateral lesions (14/54, 25.9%). The mean lesion size was 15.0 ± 7.8 mm (range 5.5–42.9 mm). Based on the reference standard of adenomas, we recognized 34 adrenal lesions as adrenal adenomas: (1) pathologically confirmed (*n* = 20); (2) without pathological results but with TNC attenuation ≤ 10 HU (*n* = 12); and (3) without pathological results and with TNC attenuation > 10 HU, but a stable tumor size for at least 6 months at prior examinations and follow-up examinations (< 5% change in maximum diameter of the lesion), and endocrine hormone examination excluded pheochromocytoma and adrenocortical carcinoma; absence of history of malignancy excluded metastasis (*n* = 2). Of the 34 non-adenomas, 3 were pathologically confirmed as pheochromocytoma, and 31 were clinically diagnosed as adrenal hyperplasia. The demographics and clinical characteristics of all lesions and subgroups (adenomas and non-adenomas) are shown in Table 1.

**Table 1:** Demographics and clinical characteristics of all lesions and in subgroups

Characteristics	All lesions (68 lesions in 54 participants)	Subgroups		*p*-value
		Adenomas (34 lesions in 30 participants)	Non-adenomas (34 lesions in 31 participants)	
Age (mean ± SD)	45.3 ± 13.2	45.9 ± 10.9	44.6 ± 14.4	0.689
Gender (*n*)	27 females	16 females	14 females	0.523
Lesion size (mm)	15.0 ± 7.8	15.5 ± 7.2	14.5 ± 8.4	0.595
CT attenuation of 2D measurements				
TNC (HU)	18.0 ± 14.4	7.8 ± 10.1	28.2 ± 10.1	< 0.001
VNC_Conv_ (HU)	26.0 ± 12.6	18.7 ± 9.9	33.3 ± 10.8	< 0.001
VNC_PC_ (HU)	23.7 ± 13.2	16.4 ± 10.0	31.1 ± 12.0	< 0.001
CT attenuation of 3D measurements				
TNC (HU)	17.3 ± 13.0	8.6 ± 9.0	26.0 ± 10.2	< 0.001
VNC_Conv_ (HU)	26.1 ± 10.9	19.1 ± 8.6	33.0 ± 8.3	< 0.001
VNC_PC_ (HU)	24.2 ± 11.3	17.4 ± 8.9	31.0 ± 9.1	< 0.001

*2D* Two-dimensional, *3D* Three-dimensional, *TNC* True noncontrast, *VNC* Virtual noncontrast, *VNC*_*Conv*_ Conventional VNC, *VNC*_*PC*_ PureCalcium VNC

### CT attenuation on TNC and VNC images

**Fig. 3:**
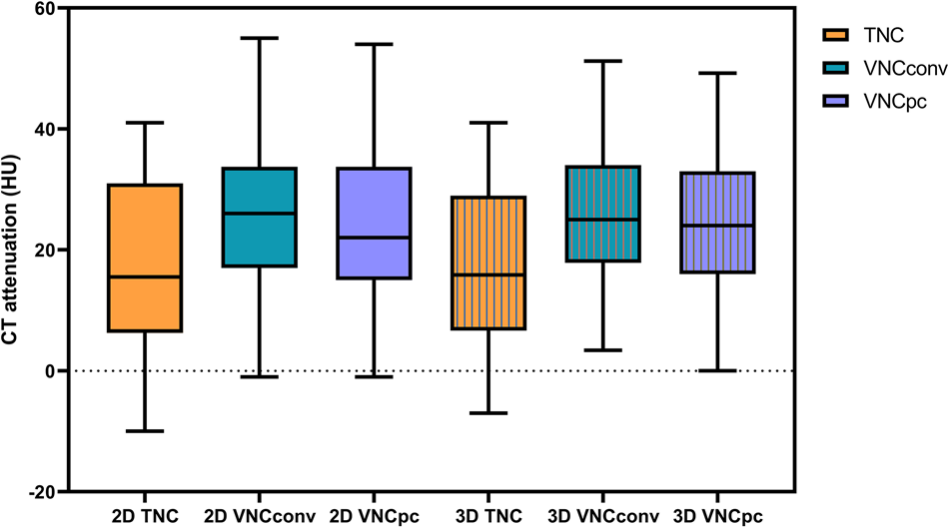
CT attenuation of adrenal lesions on TNC, VNC_Conv_, and VNC_PC_ of 2D and 3D measurements. There were statistically significant differences between VNC_Conv_, VNC_PC_, and TNC for either 2D or 3D measurements (2D: TNC *versus* VNC_Conv_, TNC *versus* VNC_PC_, and VNC_Conv_ *versus* VNC_PC_, all *p* < 0.001; 3D: TNC *versus* VNC_Conv_ and TNC *versus* VNC_PC_, *p* < 0.001; VNC_Conv_ *versus* VNC_PC_, *p* = 0.003). CT attenuation of TNC, VNC_Conv_, and VNC_PC_ did not differ significantly between 2D and 3D measurements (2D TNC *versus* 3D TNC, *p* = 0.229; 2D VNC_Conv_ *versus* 3D VNC_Conv_, *p* = 0.919; 2D VNC_PC_ *versus* 3D VNC_PC_, *p* = 0.467). TNC, True noncontrast; VNC, Virtual noncontrast; VNC_Conv_, Conventional VNC; VNC_PC_, PureCalcium VNC

**Fig. 4:**
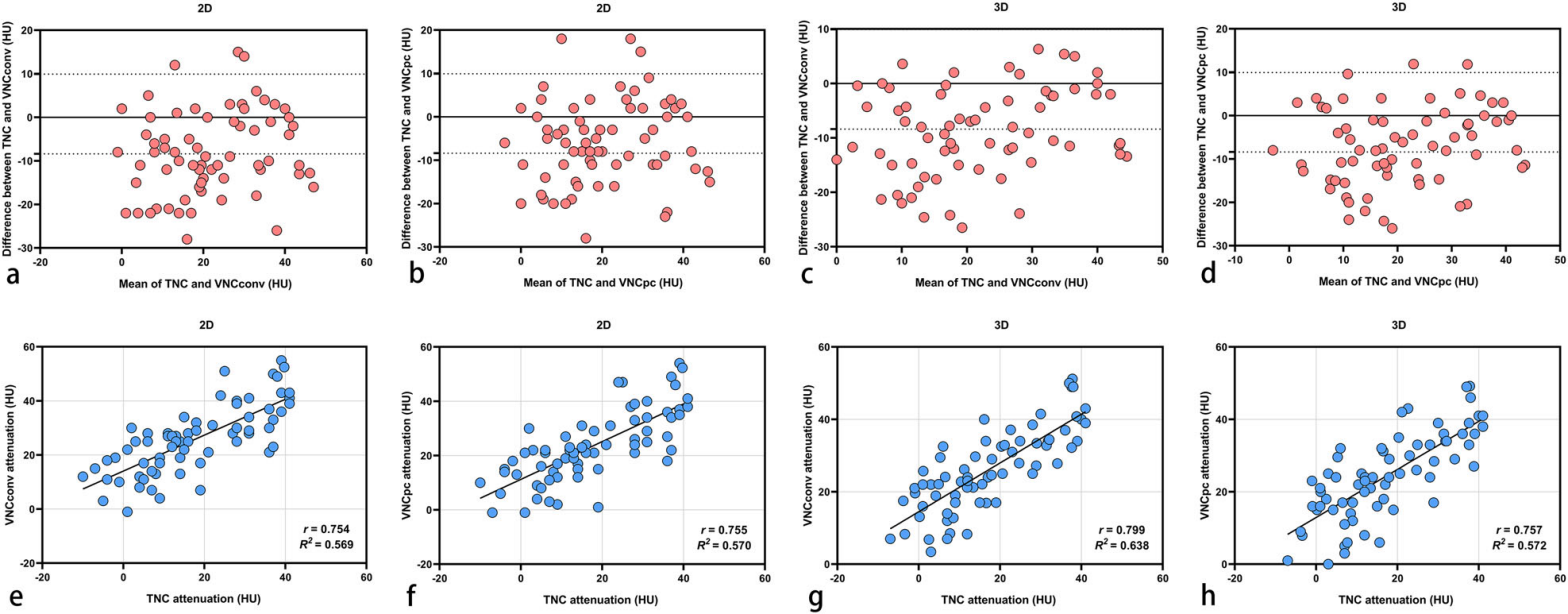
**a**–**d** Bland–Altman plots showing the agreement between TNC and VNC attenuation. Solid horizontal lines represent mean bias, and upper and lower dashed lines denote upper and lower limits of agreement, respectively. **e**–**h** There was a moderate positive correlation between TNC and VNC_Conv_ and between TNC and VNC_PC_ for 2D measurements (*r* = 0.754, *R*^*2*^ = 0.569 and *r* = 0.755, *R*^*2*^ = 0.570, respectively) and for 3D measurements (*r* = 0.799, *R*^*2*^ = 0.638 and *r* = 0.757, *R*^*2*^ = 0.572, respectively). TNC, True noncontrast; VNC, Virtual noncontrast; VNC_Conv_, Conventional VNC; VNC_PC_, PureCalcium VNC

For all lesions, CT attenuation on VNC_Conv_ (2D: 26.0 ± 12.6 HU, 3D: 26.1 ± 10.9 HU) and VNC_PC_ (2D: 23.7 ± 13.2 HU, 3D: 24.2 ± 11.3 HU) was higher than on TNC (2D: 18.0 ± 14.4 HU, 3D: 17.3 ± 13.0 HU). For 2D measurements, the mean difference between VNC and TNC was 8.0 HU for VNC_Conv_ and 5.7 HU for VNC_PC_. For 3D measurements, the mean difference between VNC and TNC was 8.8 HU for VNC_Conv_ and 6.9 HU for VNC_PC_. There were statistically significant differences between VNC_Conv_, VNC_PC_, and TNC for either 2D or 3D measurements (2D: TNC *versus* VNC_Conv_, TNC *versus* VNC_PC_, and VNC_Conv_ *versus* VNC_PC_, all *p* < 0.001; 3D: TNC *versus* VNC_Conv_ and TNC *versus* VNC_PC_, *p* < 0.001; VNC_Conv_ *versus* VNC_PC_, *p* = 0.003). CT attenuation of TNC, VNC_Conv_, and VNC_PC_ did not differ significantly between 2D and 3D measurements (2D TNC *versus* 3D TNC, *p* = 0.229; 2D VNC_Conv_ *versus* 3D VNC_Conv_, *p* = 0.919; 2D VNC_PC_ *versus* 3D VNC_PC_, *p* = 0.467) (Figs. 3 and 4a–d). There was a moderate positive correlation between TNC and VNC_Conv_ and between TNC and VNC_PC_ for 2D measurements (*r* = 0.754, *R*^*2*^ = 0.569 and *r* = 0.755, *R*^*2*^ = 0.570, respectively) and for 3D measurements (*r* = 0.799, *R*^*2*^ = 0.638 and *r* = 0.757, *R*^*2*^ = 0.572, respectively) (Fig. 4e–h).

### Radiomic features on TNC and VNC images

**Fig. 5:**
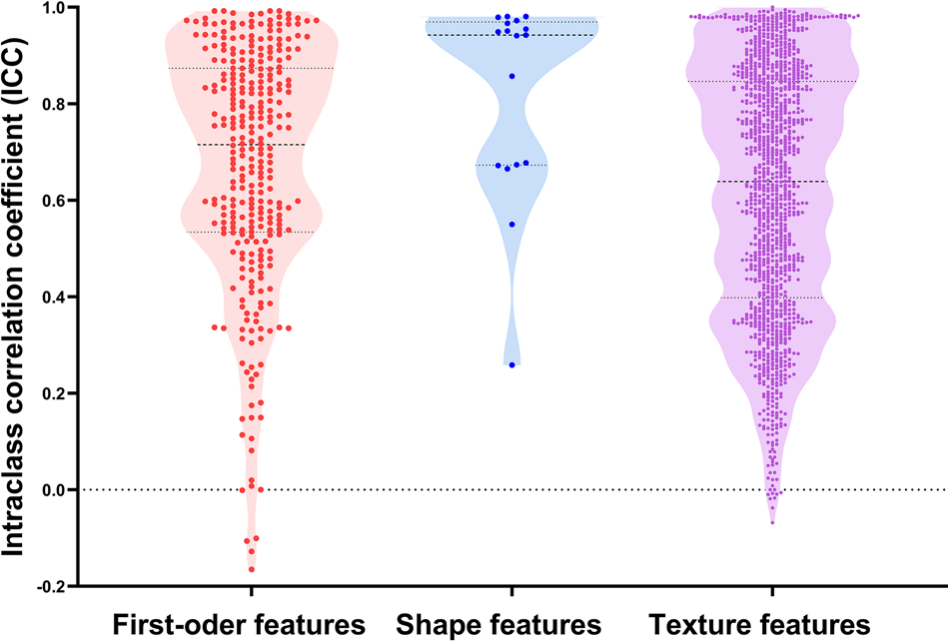
Violin plot showing the distributions of radiomic features, including first-order features, shape features, and texture features between TNC and VNC. Shape features had superior agreement, and first-order and texture features had inferior agreement. There were 117 first-order features (117/306, 38.2%), 11 shape features (11/17, 64.7%), and 405 texture features (405/1275, 31.8%) with ICC > 0.8. ICC, Intraclass correlation coefficient; TNC, True noncontrast; VNC, Virtual noncontrast

The mean ICCs of the adrenal lesion radiomic features (first-order, shape, and texture features) between TNC and VNC were 0.671, 0.822, and 0.616, respectively (Table 2). There were 117 first-order features (117/306, 38.2%), 11 shape features (11/17, 64.7%), and 405 texture features (405/1,275, 31.8%) with an ICC > 0.8, indicating excellent consistency between VNC and TNC. There was better agreement for shape features than for the other two feature types, with a greater proportion of ICC > 0.8. There was a more even distribution of the three types of features for ICCs between 0.6 and 0.8: 73 first-order features (73/306, 23.9%), 4 shape features (4/17, 23.5%), and 289 texture features (289/1,275, 22.7%) (Table 2 and Fig. 5). Following Bonferroni correction, ICCs of 1,091 radiomics features demonstrated statistically significant (all *p* < 3.13 × 10^−5^), while the remaining ICCs of 507 features were not statistically significant (all *p* ≥ 3.13 × 10^−^^5^) with ICC values below 0.493. Detailed data are shown in Supplementary Table 1 (Table S1).

**Table 2:** ICC of radiomic features between TNC and VNC images

Radiomic features	Mean ICC	ICC				Total
		> 0.8	0.6–0.8	0.4–0.6	< 0.4	
First-order features	0.671	117 (38.2%)	73 (23.9%)	73 (23.9%)	43 (14.1%)	306 (100.0%)
Shape features	0.822	11 (64.7%)	4 (23.5%)	1 (5.9%)	1 (5.9%)	17 (100.0%)
Texture features	0.616	405 (31.8%)	289 (22.7%)	261 (20.5%)	320 (25.1%)	1,275 (100.0%)

Data are mean ICC values for the three different types of features and the number of features with percentages in parentheses

*TNC* True noncontrast, *VNC* Virtual noncontrast

### Lesion size between adenomas and non-adenomas

There was no statistical difference in lesion size between adenomas (15.5 ± 7.2 mm, range 7.0–39.2 mm) and non-adenomas (14.5 ± 8.4 mm, range 5.5–42.9 mm) (*p* = 0.595). We evaluated the diagnostic performance of lesion size thresholds at 10 mm, 15 mm, and 20 mm for distinguishing adenomas from non-adenomas. The results indicated that a threshold of 10 mm yielded a sensitivity of 82.4% (28/34) and a specificity of 32.4% (11/34) for diagnosing adenomas. When the threshold was increased to 15 mm, the sensitivity and specificity were 44.1% (15/34) and 70.6% (24/34), respectively. Further increasing the threshold to 20 mm resulted in a sensitivity of 20.6% (7/34) and a specificity of 76.5% (26/34). The AUC value of lesion size in diagnosing adenomas was 0.592 (95% confidence interval [CI]: 0.454–0.729).

### CT attenuation between adenomas and non-adenomas

**Fig. 6:**
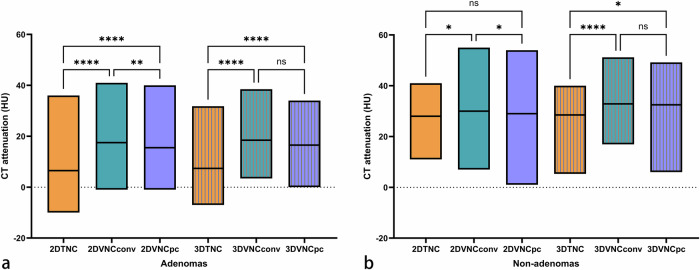
CT attenuation of adrenal adenomas (**a**) and non-adenomas (**b**) on TNC, VNC_Conv_, and VNC_PC_ of 2D and 3D measurements. TNC, True noncontrast; VNC, Virtual noncontrast; VNC_Conv_, Conventional VNC; VNC_PC_, PureCalcium VNC; ns, no significance; ^*^ *p* < 0.05; ^**^ *p* < 0.01; ^****^ *p* < 0.0001

Among the 34 adenomas, the mean CT attenuation on TNC of 2D measurements was 7.8 ± 10.1 HU, whereas the corresponding mean CT attenuation on VNC_Conv_ and VNC_PC_ were 18.7 ± 9.9 HU and 16.4 ± 10.0 HU, respectively (TNC *versus* VNC_Conv_ and TNC *versus* VNC_PC_, *p* < 0.001; VNC_Conv_ *versus* VNC_PC_, *p* = 0.002). There was a mean difference of 10.9 HU for VNC_Conv_ reconstructions and of 8.6 HU for VNC_PC_ reconstructions compared to TNC. For 3D measurements, the mean CT attenuations of adenomas were 8.6 ± 9.0 HU, 19.1 ± 8.6 HU, and 17.4 ± 8.9 HU on TNC, VNC_Conv_, and VNC_PC_ images, respectively (TNC *versus* VNC_Conv_ and TNC *versus* VNC_PC_, *p* < 0.001; VNC_Conv_ *versus* VNC_PC_, *p* = 0.078) (Fig. 6a).

Analyzing the 34 non-adenomas, the mean CT attenuation on TNC of 2D measurements was 28.2 ± 10.1 HU, whereas corresponding CT attenuations on VNC_Conv_ and VNC_PC_ were 33.3 ± 10.8 HU and 31.1 ± 12.0 HU, respectively (TNC *versus* VNC_Conv_, *p* = 0.030; TNC *versus* VNC_PC_, *p* = 0.489; VNC_Conv_ *versus* VNC_PC_, *p* = 0.026). The mean CT attenuations of non-adenomas of 3D measurements were 26.0 ± 10.2 HU, 33.0 ± 8.3 HU, and 31.0 ± 9.1 HU on TNC, VNC_Conv_, and VNC_PC_ images, respectively (TNC *versus* VNC_Conv_, *p* < 0.001; TNC *versus* VNC_PC_, *p* = 0.015; VNC_Conv_ *versus* VNC_PC_, *p* = 0.156) (Fig. 6b).

A comparison of the CT attenuation of TNC, VNC_Conv_, and VNC_PC_ between adenomas and non-adenomas revealed significant differences for both 2D and 3D measurements (all *p* < 0.001) (Table 1).

### Diagnostic performance of VNC for adenomas

**Fig. 7:**
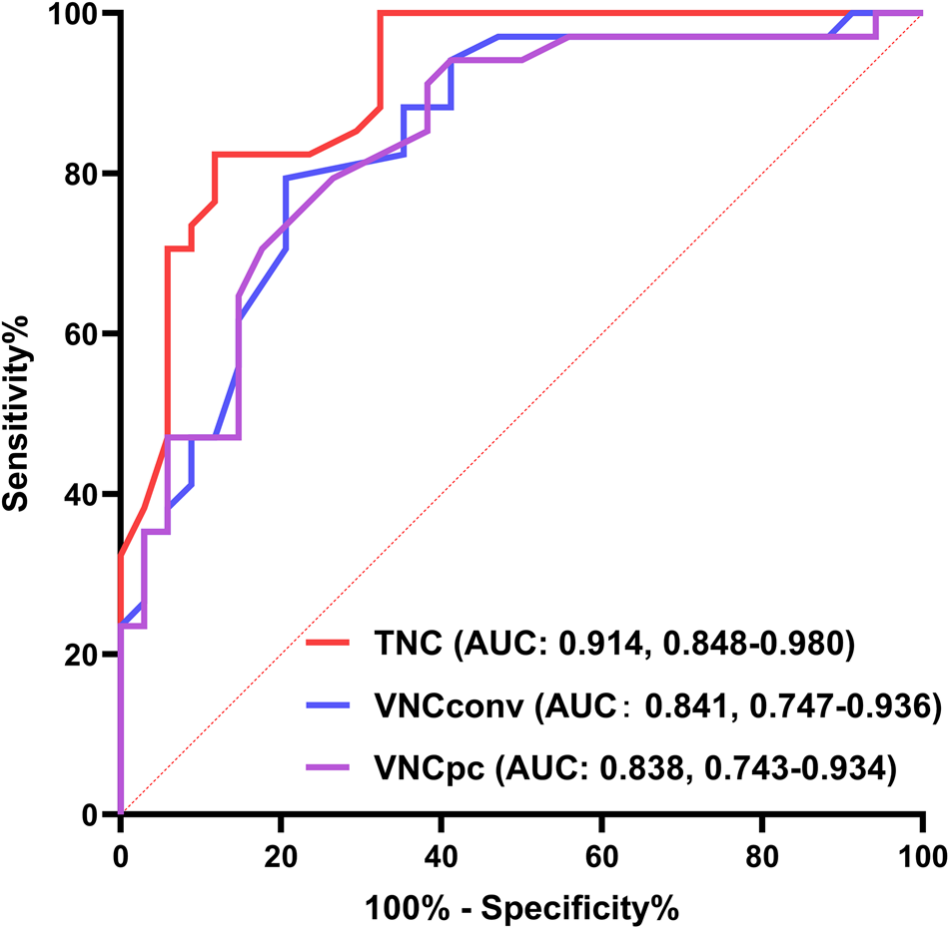
ROC curves of TNC, VNC_Conv_, and VNC_PC_ for diagnosing adenomas. The areas under the ROC curves (AUCs) in diagnosing adenoma were 0.914 for TNC, 0.841 for VNC_Conv_, and 0.838 for VNC_PC_. The Delong test showed there was no significant difference between VNC_Conv_ and VNC_PC_ in diagnosing adenomas (AUC: 0.841 *versus* 0.838, *p* = 0.873). The sensitivity and specificity for VNC_Conv_ were 79.4% and 79.4% at a clinically recommended threshold of 25 HU, respectively; the sensitivity and specificity for VNC_PC_ were 61.8% and 91.2% at a clinically recommended threshold of 20 HU. TNC, True noncontrast; VNC, Virtual noncontrast; VNC_Conv_, Conventional VNC; VNC_PC_, PureCalcium VNC

TNC showed an AUC of 0.914 (95% CI: 0.848–0.980), and the optimal threshold (maximum Youden’s index) of 17 HU was observed in our study with a sensitivity of 88.2% and a specificity of 82.4%. The AUC of VNC_Conv_ for diagnosing adenomas was 0.841 (95% CI: 0.747–0.936), and the optimal threshold (maximum Youden’s index) was 26 HU, with a sensitivity and specificity of 79.4% and 79.4%, respectively. The AUC of VNC_PC_ for diagnosing adenomas was 0.838 (95% CI: 0.743–0.934) and the optimal threshold (maximum Youden’s index) was 18 HU, with a sensitivity and specificity of 61.8% and 91.2%, respectively (Table 3 and Fig. 7). The Delong test showed there was no significant difference between VNC_Conv_ or VNC_PC_ in diagnosing adenomas (*p* = 0.873).

**Table 3:** Sensitivity and specificity of parameters for diagnosing adrenal adenomas

Parameter and threshold	Sensitivity (%)	Specificity (%)
TNC attenuation		
≤ 5 HU	44.1	100
≤ 10 HU^a^	67.7	100
≤ 15 HU^b^	82.4	82.4
≤ 17 HU^c^	88.2	82.4
≤ 20 HU	91.2	73.5
≤ 25 HU	94.1	64.7
≤ 30 HU	94.1	47.1
VNC_Conv_ attenuation		
≤ 5 HU	8.8	100
≤ 10 HU^a^	17.6	97.1
≤ 15 HU	41.2	97.1
≤ 20 HU	58.8	94.1
≤ 25 HU^b^	79.4	79.4
≤ 26 HU^c^	79.4	79.4
≤ 30 HU	88.2	47.1
VNC_PC_ attenuation		
≤ 5 HU	14.7	97.1
≤ 10 HU^a^	26.5	97.1
≤ 15 HU	50.0	94.1
≤ 18 HU^c^	61.8	91.2
≤ 20 HU^b^	61.8	85.3
≤ 25 HU	85.3	61.8
≤ 30 HU	88.2	47.1

*TNC* True noncontrast, *VNC* Virtual noncontrast, *VNC*_*Conv*_ Conventional VNC, *VNC*_*PC*_ PureCalcium VNC

^a^ Currently accepted threshold for unenhanced CT

^b^ Proposed threshold in clinical practice

^c^ Optimal threshold obtained from the maximum value of the Youden index (sensitivity + specificity − 1)

The proposed thresholds for clinical practice were 15 HU for TNC, 25 HU for VNC_Conv_, and 20 HU for VNC_PC_, with sensitivities and specificities of 82.4% and 82.4%, 79.4% and 79.4%, and 61.8% and 85.3%, respectively. Using the acknowledged threshold of 10 HU for adrenal adenomas, the sensitivities and specificities of TNC for the diagnosis of adenomas were 67.7% and 100%, those of VNC_Conv_ were 17.6% and 97.1%, and those of VNC_PC_ were 26.5% and 97.1%, respectively (Table 3).

### Diagnostic accuracy stratified by lesion size

The diagnostic performance of CT attenuation thresholds (VNC_Conv_ 25 HU, VNC_PC_ 20 HU) varied significantly across different lesion size groups (Table S2). For a threshold of 25 HU on VNC_Conv_, lesions smaller than 10 mm demonstrated optimal performance with 100% sensitivity, 81.8% specificity, and 88.2% accuracy, while lesions (≥ 10 mm and < 15 mm) maintained high sensitivity (92.3%), specificity (84.6%), and accuracy (88.5%). However, performance declined for lesions ≥ 15 mm (sensitivity, 60.0%; specificity, 70.0%, and accuracy, 64.0%). For the threshold of 20 HU on VNC_pc_, lesions smaller than 10 mm achieved perfect sensitivity (100%) and the highest specificity (90.9%), and accuracy (94.1%). The diagnostic metrics declined for larger lesions, with the accuracy of 76.9% (≥ 10 mm and < 15 mm) (sensitivity, 76.9%; specificity, 76.9%) and 56.0% (≥ 15 mm) (sensitivity, 33.3%; specificity, 90.0%).

### *Post hoc* power analyses of the sample size

We used G*Power (version 3.1.9.7, http://www.gpower.hhu.de). Based on the mean and standard deviation of CT attenuation in the adenoma and non-adenoma groups, the power of the study was greater than 0.99 (TNC, 1.0000000; VNC_Conv_, 0.9999166; and VNC_PC_, 0.9997156), and the effect size was greater than 0.80 (TNC, 2.0198020; VNC_Conv_, 1.4092966; and VNC_PC_, 1.3308755), with a two-sided α error margin of 0.05. Based on these values, the sample size was deemed adequate.

## Discussion

We compared the differences in CT attenuation values and radiomic features between VNC and TNC images of adrenal lesions on PCCT. We found that VNC reconstructions of PCCT overestimated CT attenuation of adrenal lesions compared to TNC images. CT attenuation on VNC_PC_ images was closer to that on TNC images than on VNC_Conv_ images, for both 2D and 3D measurements. TNC, VNC_Conv_, and VNC_PC_ attenuation did not differ significantly between 2D and 3D measurements. The agreement between TNC and VNC images was excellent for shape features, and there was general agreement for first-order and texture features. The diagnostic performance of TNC images for adenomas was superior to that of VNC images. The proposed thresholds (25 HU for VNC_Conv_ and 20 HU for VNC_PC_) had better diagnostic performance for discriminating adenomas from non-adenomas than the established 10 HU threshold.

Regarding the overestimation of VNC reconstructions from PCCT, few previous studies have investigated the performance of VNC from PCCT for adrenal lesions, and our findings were consistent with those of Bette et al [15]. Lennartz et al [14] concluded that VNC images over- and underestimated attenuation compared to TNC images for the assessment of adrenal adenomas. In our study, although it was uncertain whether the VNC attenuation per case was overestimated or underestimated compared with TNC attenuation, for the majority of cases and for the mean value overall, the adrenal lesion attenuation was found to be overestimated. Similarly, previous VNC studies with DECT have also yielded results that overestimate the attenuation of adrenal lesions [6–10]. Some scholars pointed out that this is likely because the residual iodine was not completely subtracted [6, 16, 17]. Additionally, the correlation between VNC and TNC in this study was moderate and lower than that observed in a previous DECT study (0.75 *versus* 0.92) [6].

Overestimation of adenoma attenuation by VNC leads to a reduction in the sensitivity of VNC in the diagnosis of adenomas [6, 7], thereby requiring a higher threshold for clinical diagnosis. In a study using PCCT, 10 of 36 adenomas were misclassified when applying the 10 HU threshold to VNC (seven misclassified as lipid-rich, three misclassified as lipid-poor) [14]. Bette et al proposed a higher threshold of 26 HU for VNC_Conv_ reconstructions with a sensitivity of 86.7% and a specificity of 75.6%, for more accurate VNC identification of adrenal adenomas and metastases than by using the established 10 HU threshold [15]. Using the threshold of 26 HU, our study had a lower sensitivity and higher specificity compared with the previous study. This may be attributed in part to the fact that the non-adenomas included in our study encompassed adrenal hyperplasia and pheochromocytoma, but did not include metastases, and differences in disease types could potentially lead to an underestimated detection rate of adrenal adenomas. Using 10 HU as the threshold, the sensitivity and specificity of TNC in this study were 67.7% and 100%, which were slightly different from those of prior studies (71% and 98% [3], 79% and 96% [4]). We believe that differences in inclusion criteria and disease types within groups contributed to the slightly different results.

A meta-analysis revealed that VNC images generated from DECT exhibited comparable sensitivity to TNC images for the diagnosis of adenomas [18]. While TNC images demonstrated superior diagnostic performance for adenomas compared to VNC images in our study, the role of VNC is nonetheless significant. Adrenal lesions are usually found incidentally on contrast-enhanced CT scans. However, incidental adrenal masses may not be identified as adenomas due to significant overlap in attenuation on routine single-phase contrast-enhanced CT. Therefore, further imaging or follow-up is required, which will lead to increased radiation exposure and medical costs [15]. VNC images derived from contrast-enhanced DECT serve as a valuable alternative for diagnosing adenomas, potentially reducing the need for additional examinations [2]. Given the convenience of clinical application, we recommend using thresholds of 25 HU for VNC_Conv_ and 20 HU for VNC_PC_ as more general thresholds. It should be noted that this study is preliminary and based on a small sample size. Therefore, further research with a larger sample is necessary to validate these findings.

Lesion size significantly impacted the diagnostic accuracy of CT attenuation thresholds, with optimal performance observed in small lesions. The threshold of 25 HU on VNC_Conv_ was more reliable for lesions under 15 mm, while the threshold of 20 HU on VNC_pc_ demonstrated superior performance for lesions under 10 mm. These findings suggest that the proposed thresholds are optimal for small lesions (VNC_Conv_ 25 HU for lesions < 15 mm, VNC_pc_ 20 HU for lesions < 10 mm) to maintain diagnostic accuracy in clinical practice.

In this study, image reconstruction was performed using both the VNC_Conv_ algorithm and the PureCalcium algorithm. Although VNC_PC_ is designed to preserve calcification, our study demonstrated that it exhibited superior VNC imaging capabilities compared to the VNC_Conv_ algorithm, with smaller CT attenuation differences from TNC. These findings were consistent with those reported by Bette et al [15]. Recent studies that analyzed this algorithm showed more consistent VNC attenuation with TNC attenuation for both calcified lesions and soft tissue [19–21].

The results of CT attenuation indicated no statistically significant difference between 2D and 3D measurements of the TNC, VNC_Conv_, and VNC_PC_ images. This finding suggested that it may be more efficient to select the largest slice to measure CT attenuation rather than the entire volume. This approach could potentially greatly reduce the human resources and time required for analysis.

Furthermore, a comparative analysis of the radiomic features showed that VNC had superior agreement for shape features but inferior agreement for first-order and texture features. This suggests that VNC can simulate the shape of lesions relatively accurately, but further improvements are needed to process more complex and detailed information hidden in the greyscale images. Notably, the “original_firstorder_Mean” parameter among the radiomic features, which reflected a mean CT attenuation of the entire lesion, had an ICC of 0.832. However, several other features relevant to CT attenuation, such as “original_firstorder_Maximum”, “original_firstorder_Minimum”, “original_firstorder_MeanAbsoluteDeviation”, and “original_firstorder_Range”, had general agreements of 0.594, 0.597, 0.515, and 0.647, respectively. This may suggest that the CT attenuation of the lesions in this study covered a wide range, and there was significant heterogeneity within the lesions, leading to disparities in the results of the macroscopic CT attenuations. In future studies, when constructing diagnostic models using radiomic features, researchers should focus more on the features with higher consistencies and avoid using features with ICC values below 0.5.

There are some limitations to our study. First, this was a single-center prospective study and may have a bias in participant selection. Second, some adenomas were diagnosed not by histopathology, but by further imaging (*e.g*., unenhanced CT) or follow-up. Third, the sample size of this study was relatively small, but the post-hoc power analysis proved the sample size was adequate. Further studies with a larger sample size are needed. Lastly, some radiomics features did not conform to the image biomarker standardization initiative and may affect comparability.

In conclusion, VNC reconstructions of PCCT overestimated the CT attenuation compared to TNC images. The AUC values in diagnosing adenoma were 0.914 for TNC, 0.841 for VNC_Conv_, and 0.838 for VNC_PC_. The proposed thresholds of 25 HU for VNC_Conv_ and 20 HU for VNC_PC_ demonstrated improved sensitivities and specificities over the established 10 HU threshold.

## Supplementary information

Supplementary information

Supplementary Information

## Data Availability

All data generated or analyzed during this study are included in this published article and its supplementary information files.

## Abbreviations

2D: Two-dimensional
3D: Three-dimensional
AUC: Area under the ROC curve
CI: Confidence interval
CT: Computed tomography
DECT: Dual-energy CT
ICC: Intraclass correlation coefficient
PCCT: Photon-counting CT
ROC: Receiver operating characteristic
TNC: True noncontrast
VNC: Virtual noncontrast
VNC_Conv_: Conventional VNC
VNC_PC_: PureCalcium VNC
