# Supplementary material for: Virtual noncontrast images of adrenal lesions: a photon-counting CT prospective study
Source: Eur Radiol Exp. 2025 Aug 29;9:82. doi: 10.1186/s41747-025-00621-x (PMC12396999; doi:10.1186/s41747-025-00621-x)
Supplement: Supplementary file 1 — Supplementary information [file 41747_2025_621_MOESM1_ESM.pdf]

Virtual noncontrast images of adrenal lesions: a photon-counting CT prospective study

ELECTRONIC SUPPLEMENTARY MATERIAL

Table S1 Mean intraclass correlation coefficient (ICCs) of the adrenal lesion radiomic features between true non-contrast (TNC) and virtual non-contrast (VNC).

Please see Table S1 in a separate supplemental file.

Table S2 Diagnostic performance of CT attenuation thresholds stratified by lesion size

Lesion size	VNC_{Conv} (≤ 25 HU)			VNC_{PC} (≤ 20 HU)		
	Sensitivity	Specificity	Accuracy	Sensitivity	Specificity	Accuracy
size <10 mm	100% (6/6)	81.8% (9/11)	88.2% (15/17)	100% (6/6)	90.9% (10/11)	94.1% (16/17)
10 mm ≤ size < 15 mm	92.3% (12/13)	84.6% (11/13)	88.5% (23/26)	76.9% (10/13)	76.9% (10/13)	76.9% (20/26)
size ≥ 15 mm	60.0% (9/15)	70.0% (7/10)	64.0% (16/25)	33.3% (5/15)	90.0% (9/10)	56.0% (14/25)

VNC, virtual non-contrast; VNC_{Conv}, conventional VNC; VNC_{PC}, PureCalcium VNC
